# Protein structure alignment significance is often exaggerated

**DOI:** 10.1101/2025.07.17.665375

**Published:** 2025-07-19

**Authors:** Robert C. Edgar, Harutyun Sahakyan

## Abstract

Machine learning has generated millions of high-quality predicted protein structures, creating a need for computationally efficient structure search algorithms and robust estimates of statistical significance at this scale. We show that unrelated proteins have a universal tendency towards convergent evolution of secondary and tertiary motifs, causing an excess of high-scoring false positive alignments. To address this excess, and to accommodate recent innovations in search algorithm design, we describe a novel method for estimating statistical significance. We implement our approach in Reseek, showing that its *E* -values are accurate, scale successfully with database size, and are robust against the (generally unknown) diversity of folds in the database. We investigate popular structure search and alignment algorithms, finding that previous methods routinely overestimate significance by up to six orders of magnitude.

## Full Text Availability

The license terms selected by the author(s) for this preprint version do not permit archiving in PMC. The full text is available from the preprint server.

